# Endoscopic Ultrasound Findings in Patients Diagnosed with Exocrine Pancreatic Insufficiency by Low Fecal Elastase-1

**DOI:** 10.1155/2019/5290642

**Published:** 2019-08-14

**Authors:** Mazen Shobassy, Nedaa Husainat, Abdalaziz Tabash, Kalpesh Patel, Hashem B. El-Serag, Mohamed O. Othman

**Affiliations:** ^1^Section of Gastroenterology and Hepatology, Department of Medicine, Baylor College of Medicine, Houston, TX, USA; ^2^Center for Innovations in Quality, Effectiveness, and Safety, Michael E. DeBakey Veterans Affairs Medical Center, Houston, TX, USA; ^3^Section of Gastroenterology and Hepatology, Michael E. DeBakey Veterans Affairs Medical Center, Houston, TX, USA

## Abstract

**Background and Aims:**

Fecal elastase-1 (FE-1) as a screening test for exocrine pancreatic insufficiency (EPI) is gaining popularity in clinical practice. The role of imaging in patients with FE-1-related suspicion of EPI remains unclear. The aim of this study was to characterize endoscopic ultrasound (EUS) findings for patients with low FE-1.

**Methods:**

A retrospective cross-sectional study was performed in 40 patients who had low FE-1 and underwent EUS to evaluate the pancreas. We obtained data on demographic and lifestyle factors, EUS findings, and histopathology results. We compared these variables between patients with FE‐1 < 100 mcg/g vs. 100-200 mcg/g.

**Results:**

Most patients (82.5%) established one or more new diagnoses from EUS. Diagnoses included: definitive chronic pancreatitis (*n* = 29, 72.5%), fatty pancreas (*n* = 9, 22.5%), and pancreatic solid mass or cyst (*n* = 9, 22.5%). Half (*n* = 4) of the solid or cystic lesions were neoplastic. All patients with a solid pancreatic mass also had concurrent chronic pancreatitis. There were no significant differences in EUS findings or demographic or lifestyle factors between groups with FE‐1 < 100 mcg/g vs. 100-200 mcg/g.

**Conclusion:**

Chronic pancreatitis is the most common EUS finding in patients with low FE-1 levels. EUS appears helpful in determining the cause of EPI in most patients with low FE-1 and may detect unsuspected pancreatic neoplasia.

## 1. Introduction

Exocrine pancreatic insufficiency (EPI) is characterized by diarrhea, bloating, abdominal pain, and weight loss [1]. Complications of EPI include cardiovascular disease, compromised immunity, psychological disorders, bleeding disorders, night blindness, and muscle weakness [2–4]. However, EPI can be asymptomatic [5] and unrecognized in the early stages where diagnosis is challenging.

There are several diagnostic tests for EPI, but all have limitations [1, 6, 7]. Direct tests of pancreatic function, such as secretin-cholecystokinin or secretin-cerulein stimulation tests, have the highest accuracy for evaluating pancreatic secretions. However, their use is limited by high expense and lack of standardization of testing. Indirect tests such as 72-hour fecal fat quantification are considered a gold standard for diagnosing steatorrhea, but are cumbersome and have poor sensitivity for diagnosing mild to moderate EPI. Furthermore, many of these tests are inaccurate for patients who are taking pancreatic enzyme replacement therapy [1, 6, 7].

Fecal elastase-1 (FE-1) is a widely available noninvasive diagnostic tool for EPI. Elastase-1 is an enzyme produced by pancreatic acinar cells. Because of minimal degradation as it passes through the gut, fecal levels of elastatse-1 correlate well with pancreatic enzyme output [8, 9]. A recent meta-analysis by Vanga et al. found that FE-1 had a pooled sensitivity of 0.96 and specificity of 0.88 for identifying patients with EPI compared to quantitative fecal fat estimation [10]. With a relatively high sensitivity and wide availability, FE-1 is more frequently used to test for and diagnose possible EPI cases.

Chronic pancreatitis is the most common cause of EPI, but other etiologies such as cystic fibrosis and pancreatic neoplasms can also be present [6, 11–13]. Emerging data has also associated pancreatic steatosis with EPI [14]. Once EPI is diagnosed, patients are frequently started on a therapeutic/diagnostic trial of pancreatic enzyme replacement. Though symptoms may improve after starting enzyme replacement, it remains unclear if pancreatic imaging is still useful on the initial evaluation of EPI.

Endoscopic ultrasound (EUS) is highly sensitive for the diagnosis of pancreatic neoplasms, chronic pancreatitis, and fatty pancreas [15–17]. However, the role of EUS in the management of patients with suspected EPI is unclear. There are no data on EUS findings in patients with EPI detected by low FE-1 levels. The aim of this study is to examine the EUS findings in patients with low FE-1, without prior diagnoses of chronic pancreatitis, cystic fibrosis, or pancreatic neoplasms.

## 2. Methods

This study was approved by the Institutional Review Board at Baylor College of Medicine. We conducted a cross-sectional study among patients seen at Baylor Clinic in Houston, Texas, from January 2015 to December 2016. Consecutive patients who were 18 years of age or older referred for suspected EPI, as defined by a FE‐1 < 200 mcg/g, and underwent EUS were included in the study. We excluded pregnant patients and those with established cystic fibrosis because these patients already had a known cause of EPI. We collected data on patient demographics (gender, age), anthropometrics (weight, height), lifestyle factors (smoking history, alcohol use), and pancreatic testing/imaging (FE-1, CT, MRI, EUS findings).

### 2.1. Fecal Elastase-1

In all patients, FE-1 test was determined using a commercial kit (LabCorp, pancreatic elastase-1, United States) that employs enzyme-linked immunosorbent assay (ELISA) of monoclonal antibody-based detection system specific for pancreatic elastase-1. Based on lab references, normal FE-1 level was greater than 200 mcg/g. Moderate EPI was defined as 100-200 mcg/g, and severe EPI was defined as less than 100 mcg/g.

### 2.2. EUS Findings

EUS procedures were performed by one of three expert endosonographers (each previously performed more than 1000 EUS procedures). EUS was performed using an Olympus (Olympus Medical Systems, Tokyo, Japan) or Pentax (Pentax Medical Company, Montvale, NJ, USA) radial or curved-linear arrayed scope. All patients were placed in the left lateral position, and using radial arrayed EUS, the head of the pancreas was examined through the duodenum. The pancreatic body to tail was scanned through the stomach. Using curved-linear arrayed EUS, the head of the pancreas was scanned through the duodenum and stomach.

We reviewed the EUS findings for parenchymal changes (including hyperechoic foci, hyperechoic strands, lobularity, and cysts) and ductal changes (including hyperechoic ductal margins, dilated main pancreatic duct, duct irregularity, dilated side branches, or intraductal stones) as well as pancreatic calcifications, atrophy, or masses. Chronic pancreatitis was defined according to the Rosemont classification which included major A and B features [18].

Irrespective of chronic pancreatitis, patients with diffuse echogenicity throughout the pancreas were considered to have a fatty pancreas. If pancreatic masses or cysts were detected on EUS, fine needle aspiration and/or biopsy was performed. Cytology and histological reports were reviewed for final diagnosis.

### 2.3. Statistical Analysis

We stratified patients based on severe (FE‐1 < 100 mcg/g) or moderate (100-200 mcg/g) EPI. EUS findings were aggregated as definite or indeterminate chronic pancreatitis as 2 mutually exclusive categories and fatty pancreas, cystic lesions, or pancreatic mass as not mutually exclusive. We compared the two FE-1 groups with respect to demographics, anthropometrics, lifestyle factors, and EUS pancreatic findings. Distributions of these variables between EPI patient groups were analyzed using *t*-test and the chi-square test.

## 3. Results

### 3.1. Study Characteristics

A total of 40 patients with a FE‐1 < 200 mcg/g and EUS were included in the study (21 patients had a FE‐1 < 100 mcg/g, 19 patients had a FE-1 of 100-200 mcg/g). The study population characteristics are shown in Table 1. Overall, 16 male and 24 female patients were included. Documented history of smoking was reported in 24 of 40 patients, and 15 patients had a history of heavy alcohol use. The average age of patients was 54.0 years (standard deviation of 13.0 years) with a range of 25-80 years. The mean body mass index (BMI) was 26.4 ± 5.2 kg/m^2^. Presenting symptoms such as diarrhea, reported weight loss, and history of diabetes were also included. There were no statistically significant differences in demographic, lifestyle factors, or presenting symptoms between the FE‐1 < 100 mcg/g and 100-200 mcg/g groups.

### 3.2. EUS Findings

Most patient (33/40, 82.5%) were found to have one or more new diagnoses based on EUS findings. 14 of 40 patients (35.0%) had 2 or more diagnoses. These diagnoses were definitive chronic pancreatitis (72.5%), fatty pancreas (22.5%), and a cyst or mass (22.5%) (Table 2).

#### 3.2.1. Chronic Pancreatitis

Definitive chronic pancreatitis was found in 29 of 40 patients (72.5%). Among patients with severe EPI (FE‐1 < 100 mcg/g), 17/21 (81.0%) had definitive chronic pancreatitis, compared to 12/19 (63.2%) of patients with moderate EPI (FE‐1 = 100-200 mcg/g) (*p* = 0.21). Only 2 patients had a normal appearing pancreas on EUS, and both had a FE‐1 < 100 mcg/g. Indeterminate chronic pancreatitis on EUS was found in 7 of 40 patients (17.5%); the distribution was 2/21 (9.5%) among patients with FE‐1 < 100 mcg/g and 5/19 (26.3%) among those with FE-1 between 100 and 200 mcg/g (*p* = 0.16). Specific endoscopic ultrasound findings were also compared between groups and has been listed in Table 3. Patients with FE‐1 < 100 mcg/g had a significantly higher prevalence of pancreatic duct dilation, pancreatic calcification, or atrophy of the pancreas.

#### 3.2.2. Fatty Pancreas

A total of 9/40 patients (22.5%) had a fatty pancreas on EUS. Seven of these patients additionally had features of definite (*n* = 6) or indeterminate chronic pancreatitis (*n* = 1). When stratified by FE-1 levels, 4/21 (19.0%) with a FE‐1 < 100 mcg/g and 5/19 (26.3%) with FE‐1 = 100-200 mcg/g had a fatty pancreas on EUS (*p* = 0.30).

#### 3.2.3. Pancreatic Cyst or Mass

Overall, 9/40 patients (22.5%) had a solid pancreatic mass (*n* = 6) or cyst (*n* = 3) seen on EUS. All patients with a solid mass also had concurrent chronic pancreatitis. The average size of all lesions was 16 mm, and 6 of 9 patients had a lesion < 20 mm. Seven of 9 patients had cross-sectional imaging prior to EUS, but only 2/7 patients (29%) had the same EUS-detected lesion seen on prior imaging modalities. All cross-sectional imaging consisted of triple phase CT scan or MRI of the abdomen performed at our facility. FNA and/or FNB was performed on all lesions detected during EUS. Final cytopathology and surgical pathology of solid lesions revealed one pancreatic adenocarcinoma, one neuroendocrine tumor, one leiomyoma, and three chronic pancreatitis. The 3 cystic lesions were intraductal papillary mucinous neoplasm, walled-off pancreatic necrosis, and a simple cyst (Table 4).

## 4. Discussion

As more patients undergo screening for EPI, evaluations of patients with low levels of FE-1 will increase. Although chronic pancreatitis is the most common cause of EPI, other diseases such as pancreatic cancer are also potential etiologies. The role of imaging in this population is unclear, and this study is the first to characterize the EUS findings in these patients.

We found that among 40 patients who presented with low FE-1 and underwent EUS, 82.5% established at least one new diagnosis (definitive chronic pancreatitis, fatty pancreas, mass, or cyst) from EUS. 72.5% were diagnosed with definite chronic pancreatitis, and 17.5% had probable chronic pancreatitis. Irrespective of chronic pancreatitis, EUS also showed cyst/mass (22.5%) or a fatty pancreas (22.5%). We also compared EUS findings in patients with FE‐1 < 100 mcg/g vs. 100-200 mcg/g, and while the proportion of patients with abnormal EUS findings was higher in severe than moderate FE-1 defined EPI, these differences were not statistically significant. Overall, only two patients (5.0%) had a normal appearing pancreas on EUS. These findings suggest that EUS has a high diagnostic yield in this patient population and therefore should be considered as follow-up tests among most patients with abnormally low FE-1.

A large proportion (9/40, 22.5%) of all patients in this study also had a cyst or mass seen on EUS. Of the patients with a solid pancreatic mass, all had concurrent chronic pancreatitis. Three of these lesions were neoplasms that were sampled at the time of the initial EUS. The lesions were often small, and only 29% of these lesions were seen on other imaging modalities. These findings are consistent with other studies that suggest CT and MRI are less sensitive for lesions < 2 cm in size [19] and further highlight the higher risk for pancreatic neoplasms in patients with chronic pancreatitis [20–23]. Early EUS evaluation in patients with low FE-1 levels may hasten the diagnosis of chronic pancreatitis, and possibly neoplasia. We would emphasize that some of the findings in this population (small mass or cyst) are not thought to be the cause for pancreatic insufficiency, but instead, have a high prevalence in such disease.

This study has the inherent limitations of a cross-sectional study design. It only examines the prevalence of disease at a specific point in time and does not give insight to incidence of disease. The prevalence of diseases of long duration and low fatality rate, such as chronic pancreatitis, may also be overestimated due to this study design. Given the cross-sectional study design and no control group, it is not possible to conclude that any of the study outcomes were the cause for the abnormal FE-1 in these patients. The study was limited by its relatively small sample size especially when evaluating the difference between groups. Large multicenter studies are needed to evaluate if these results can be further generalized to other populations.

## Figures and Tables

**Table 1:** Demographic, anthropometric, lifestyle factors, and presenting symptoms of study population.

	Fecal elastase-1 levels			
	Overall	Severe EPI (<100 mcg/g)	Moderate EPI (100-200 mcg/g)	*p* value
Male	40.0% (16/40)	52.4% (11/21)	26.3% (5/19)	0.09
Age (year)	54.0 ± 13.0	54.6 ± 12.2	53.4 ± 14.1	0.78
BMI (kg/m^2^)	26.4 ± 5.2	26.6 ± 5.0	26.3 ± 5.4	0.87
History of tobacco smoking	60.0% (24/40)	47.6% (10/21)	73.7% (14/19)	0.09
History of alcohol abuse	37.5% (15/40)	42.9% (9/21)	31.6% (6/19)	0.46
Reported diarrhea	85.0% (36/40)	85.7% (18/21)	84.2% (16/19)	0.89
Reported weight loss	47.5% (19/40)	52.4% (11/21)	42.1% (8/19)	0.52
Diabetes	27.5% (11/40)	38.1% (8/21)	15.8% (3/19)	0.11

**Table 2:** Diagnostic findings of endoscopic ultrasound in patients with FE‐1 < 200 mcg/g.

	Fecal elastase-1 levels			
	Overall	Severe EPI (<100 mcg/g)	Moderate EPI (100-200 mcg/g)	*p* value
Chronic pancreatitis				
Definite	72.5% (29/40)	81.0% (17/21)	63.2% (12/19)	0.21
Indeterminate	17.5% (7/40)	9.5% (2/21)	26.3% (5/19)	0.16
Fatty pancreas	22.5% (9/40)	19.0% (4/21)	26.3% (5/19)	0.30
Pancreatic mass	15.0% (6/40)	19.0% (4/21)	10.5% (2/19)	0.45
Pancreatic cystic lesion	7.5% (3/40)	4.8% (1/21)	10.5% (2/19)	0.49

**Table 3:** Comparison of endoscopic ultrasound findings in patients with FE‐1 < 200 mcg/g.

EUS findings	Fecal elastase-1 levels			
	Overall	Severe EPI (<100 mcg/g)	Moderate EPI (100-200 mcg/g)	*p* value
Hyperechoic strand	87.5% (35/40)	81.0% (17/21)	94.7% (18/19)	0.22
Hyperechoic foci	82.5% (33/40)	81.0% (17/21)	84.2% (16/19)	0.79
Lobulation	70.0% (28/40)	76.2% (16/21)	63.2% (12/19)	0.37
Dilated duct	37.5% (15/40)	52.4% (11/21)	21.1% (4/19)	0.04
Visible side branch	52.5% (21/40)	61.9% (13/21)	42.1% (8/19)	0.22
Hyperechoic duct wall	60.0% (24/40)	66.7% (14/21)	52.6% (10/19)	0.37
Calcification	35.0% (14/40)	52.4% (11/21)	15.8% (3/19)	0.02
Atrophy	20.0% (8/40)	33.3% (7/21)	5.3% (1/19)	0.05

**Table 4:** Patients with pancreatic mass or cystic lesion on endoscopic ultrasound. Findings on EUS, histology, and cross-sectional imaging.

	Size (mm) on EUS	Histological diagnosis (based on EUS-guided FNA)	Cross-sectional imaging before EUS
*Pancreatic mass*			
1	25	Adenocarcinoma	Not appreciated
2	30	Leiomyoma	Not appreciated
3	15	Chronic pancreatitis	Not appreciated
4	15	Chronic pancreatitis	Not appreciated
5	7	Neuroendocrine tumor	Not appreciated
6	9	Chronic pancreatitis	n/a
*Pancreatic cyst*			
1	20	WOPN	Yes, reported
2	15	Simple cyst	Yes, reported
3	11	IPMN	n/a

WOPN = walled-off pancreatic necrosis; IPMN = intraductal papillary mucinous neoplasm.

## Data Availability

The data used to support the findings of this study are restricted by the Baylor College of Medicine Institutional Review Board (IRB) in order to protect patients' privacy. Data are available from Baylor College of Medicine IRB for researchers who meet the criteria for access to confidential data.
